# Identification of Modules With Similar Gene Regulation and Metabolic Functions Based on Co-expression Data

**DOI:** 10.3389/fmolb.2019.00139

**Published:** 2019-12-13

**Authors:** Edgardo Galán-Vásquez, Ernesto Perez-Rueda

**Affiliations:** ^1^Departamento de Ingeniería de Sistemas Computacionales y Automatización, Instituto de Investigaciones en Matemáticas Aplicadas y en Sistemas, Ciudad Universitaria, Universidad Nacional Autónoma de México, Ciudad de México, Mexico; ^2^Instituto de Investigaciones en Matemáticas Aplicadas y en Sistemas, Universidad Nacional Autónoma de México, Unidad Académica Yucatán, Mérida, Mexico; ^3^Centro de Genómica y Bioinformática, Facultad de Ciencias, Universidad Mayor, Santiago, Chile

**Keywords:** transcription factors, gene expression, metabolism, gene co-expression networks, WGCNA

## Abstract

Biological systems respond to environmental perturbations and to a large diversity of compounds through gene interactions, and these genetic factors comprise complex networks. In particular, a wide variety of gene co-expression networks have been constructed in recent years thanks to the dramatic increase of experimental information obtained with techniques, such as microarrays and RNA sequencing. These networks allow the identification of groups of co-expressed genes that can function in the same process and, in turn, these networks may be related to biological functions of industrial, medical and academic interest. In this study, gene co-expression networks for 17 bacterial organisms from the COLOMBOS database were analyzed via weighted gene co-expression network analysis and clustered into modules of genes with similar expression patterns for each species. These networks were analyzed to determine relevant modules through a hypergeometric approach based on a set of transcription factors and enzymes for each genome. The richest modules were characterized using PFAM families and KEGG metabolic maps. Additionally, we conducted a Gene Ontology analysis for enrichment of biological functions. Finally, we identified modules that shared similarity through all the studied organisms by using comparative genomics.

## Introduction

Organisms are dynamic systems that respond to intracellular and extracellular signals through the regulated expression of their genes. In recent years, a large number of experiments utilizing high-throughput technologies, including microarrays and RNA sequencing (RNA-seq), have been performed to analyze this differential expression, allowing the identification of genes co-expressed in a particular condition. Recent approaches have shown that there are underlying properties that can only be explained by studying organisms as complex systems (Kitano, 2002; Trewavas, 2006). In this context, a systematic analysis to understand the gene expression in a particular genome is through Gene Co-expression Networks (GCNs), where the network *G* = (*V, E*) is composed of a set of nodes (*V*) that represent the genes and a set of edges (*E*) that indicate significant co-expression relationships (Stuart et al., 2003; Junker and Schreiber, 2008). These types of networks maintain the structural properties of real networks, such as scale-free topology, which means that there are some highly, connected nodes, namely hubs, and a large number of nodes with a small number of connections (Van Noort et al., 2004; Tsaparas et al., 2006).

In this regard, different algorithms have been developed to reconstruct GCNs; in particular, Weighted Gene Co-expression Network Analysis (WGCNA) allows the construction of networks by considering not only the co-expression patterns between two genes but also the overlapping of neighbor genes (Zhang and Horvath, 2005). Thus, highly correlated genes are clustered into large modules based on similarities in their expression profiles. These modules are often enriched for genes that share similar biological functions (Mueller et al., 2017; van Dam et al., 2018). WGCNA also compares different GCNs to identify conserved modules between species or cell types (Yang et al., 2014; Bakhtiarizadeh et al., 2018; Hosseinkhan et al., 2018). GCNs have been used to identify genes with similar expression patterns in a set of samples, allowing the prediction of gene functions at the genome level, the functional discovery of unknown genes and their associations with diseases (Carlson et al., 2006; Emilsson et al., 2008; Amar et al., 2013).

To date, two highly conserved processes between the organisms have been identified: metabolism and gene regulation (McAdams et al., 2004; Peregrín-Alvarez et al., 2009). Both processes are mediated by specific proteins; on one hand, for metabolism, enzymes catalyze the transformation of one compound to another. On the other hand, gene expression at the transcriptional level is regulated by proteins called transcription factors (TFs). In recent works, a compendium of TF families for different organisms has been identified; and other studies have revealed promiscuity of different enzymes related to metabolism. Therefore, due to the relevance of these two types of protein-encoding genes, it is important to evaluate how the gene expression patterns are distributed in functional modules.

In this study, a gene co-expression network for 17 bacterial organisms from the COLOMBOS database using WGCNA was identified. To do this, the genes were clustered into modules with similar expression patterns. These modules were exhaustively analyzed considering the repertoire of enzymes and TFs, suggesting that these proteins are involved in similar functional processes. Additionally, to determine what functional classes are overrepresented in the respective modules, an enrichment analysis was conducted. This study provides insights into how regulatory proteins and metabolic maps are expressed in different organisms.

## Materials and Methods

### Datasets

The gene expression dataset was obtained from the COLlections of Microarrays for Bacterial OrganismS (COLOMBOS) dataset and included gene expression data for 17 different bacterial organisms with 31,982 genes and 11,224 contrasts (http://colombos.net/). In brief, COLOMBOS is a compendium of data obtained from microarray and RNA-seq experiments performed under different experimental conditions. These data are further curated and normalized, considering the following principles: (1) raw intensities are preferred as data source, (2) no local background or mismatch probe correction procedures are performed, (3) quantile normalization for high-density oligonucleotide experiments are performed, and (4) logratios are created for single-channel data according to the condition contrast definitions and combined with the dual channel measurements (Moretto et al., 2016).

Thus, we analyzed with principal components analysis (PCA) the microarray compendia of each species to identify outlier samples, i.e., those samples with a substantial difference in expression value compared with other samples. In a posterior step, the dataset results were inspected via the goodSamplesgenes function of the WGCNA R package to inspect data for missing value, and for genes with zero variance, the genes and samples identified as good genes and good samples were conserved (Largfelder and Holvarth, 2008). Finally, the total number of genes and samples considered for each organism were: Ban: 5,027 genes and 53 samples; Bce: 5,200 genes and 159 samples; Bsu: 4,176 genes and 762 samples; Bth: 4,763 genes and 217 samples; Cac: 3,777 genes and 218 samples; Cje: 1,572 samples and 103 samples; Eco: 4,321 samples, and 2,415 samples; Hpy: 1,600 genes and 83 samples; Lrh: 2,731 genes and 49 samples; Mtu: 4,068 genes and 709 samples; Pae: 5,564 genes and 375 samples; Stm: 4,466 genes and 74 samples; Sfl: 3,786 genes and 23 samples; Sme: 6,218 genes and 270 samples; Spd: 1,884 genes and 40 samples; Ttj: 2,173 genes and 303 samples; and Ype: 3,730 genes and 22 samples (Table 1). The gene expression dataset for each organism is provided as Supplementary S1.

**Table 1:** Overview of dataset and co-expression modules in this study.

**Organism (KEGG ID)**	No. of samples^*^	**No. of modules**	Avg Size/SD ^**^	**No. of ORFs/% of coverage**	**No. of TFs in modules**	**No. of enzymes in modules**	Power β^***^
*B. anthracis* strain Ames (Ban)	53	6	837.83/849.14	5,508/91.27 (5,027)	333	802	12
*B. cereus* ATCC 14579 (Bce)	159	26	200/230.77	5,366/97.9 (5,200)	339	811	12
*B. subtilis* 168 (Bsu)	762	38	109.89/67.52	4,220/98.96 (4,176)	285	759	12
*B. thetaiotaomicron* VPI-5482 (Bth)	217	12	396.9/356.56	4,816/98.9 (4,763)	223	660	10
*C. acetobutylicum* ATCC 824 (Cac)	218	7	539.57/529.80	3,778/99.99 (3,777)	254	611	14
*C. jejuni* NCTC 11168 (Cje)	103	20	78.6/54.1	1,654/95.0 (1,572)	35	413	10
*E. coli* K-12 MG1655 (Eco)	2,415	58	74.5/60.49	4,600/93.9 (4,321)	335	892	14
*H. pylori* 26695 (Hpy)	83	8	200/157.18	1,600/100 (1,600)	19	350	9
*L. rhamnosus* GG (Lrh)	49	11	248.27/210.82	2,944/92.96 (2,731)	188	507	12
*M. tuberculosis* H37Rv (Mtu)	709	29	140.27/173.83	4,096/99.3 (4,068)	245	751	10
*P. aeruginosa* PAO1 (Pae)	375	20	278.2/347.78	5,570/99.9 (5,564)	468	1,002	12
*S. enterica* LT2 (Stm)	74	20	223.3/251.72	4,548/98.2 (4,466)	328	896	9
*S. flexneri* 301 (Sfl)	23	5	757.2/505.02	4,313/88.0 (3,786)	271	776	12
*S. meliloti* 1021 (Sme)	270	15	414.53/649.46	6,218/100 (6,218)	372	797	12
*S.pneumoniae* D39 (Spd)	40	9	209.33/134.51	1,911/98.59 (1,884)	98	414	8
*T. thermophilus* HB8 (Ttj)	303	11	197.54/166.66	2,173/100 (2,173)	92	523	12
*Y. pestis* C092 (Ype)	22	11	339.09/160.73	3,979/94.39 (3,756)	238	739	14

For each species, we show the final number of experiments analyzed after PCA^*^, the total number of modules identified, the average size of the modules^**^, the coverage of genes included in the modules in relation to the total number of ORFs, the total of TFs and enzymes, and the lowest possible power term where topology approximates fits a scale-free network^***^.

### Construction of Co-expression Networks

The gene co-expression networks were constructed with the WGCNA program, which allow network construction, module detection, gene selection, calculations of topological properties, and data simulation, among others (Largfelder and Holvarth, 2008). First, the scale-free topology properties of biological networks were added by calculating the power (β) using the pickSoftThereshold function, see Table 1 for the β value per organism. Then, we constructed an adjacency matrix for each bacterium, using signed correlation networks, where nodes with negative correlation are considered unconnected; as well as, the pairwise biweight midcorrelation coefficients between all genes. This correlation method was considered because it is more powerful than the Spearman and Pearson correlation methods (Song et al., 2012; Bakhtiarizadeh et al., 2018). Then, the adjacency matrix was transformed into a Topological Overlap Matrix (TOM), where a higher TOM value allowed identification of gene modules for each pair of genes with strong interconnectivity. Therefore, it was used signed correlation networks, pairwise biweight midcorrelation coefficients and β value.

Finally, the genes were clustered into modules with similar expression patterns by using the average linkage hierarchical clustering algorithm (flashClust function) and the cutreeDynamic function was used to cut the branches of the resulting dendrogram that results in the generation of gene modules. To do this, it was used 1-TOM as a distance matrix with a minimum module size equal to 20. Therefore, the modules with highly correlated eigengenes were merged, based on a minimum height of 0.25 (mergeCloseModules function). Each module was identified with a color, where the gray color is reserved for uncorrelated genes (Horvath, 2011) and discarded; whereas the rest of modules were renamed with a number (Table S1).

To perform an analysis of hubs on the modules of interest, these were exported using the exportNetworkToCytoscape function and we selected the 100 most highly correlated genes for each module. The hubs were defined as the most highly connected nodes within the module, so we calculated the degree of connectivity for each node (K), which is defined as the number of edges adjacent to each node (Junker and Schreiber, 2008) (Figure S1). A general version of all scripts were included in Supplementary S2.

### Distribution of TFs and Enzymes

For each genome, we associated the Enzyme Commission number (E.C. number) using the Kyoto Encyclopedia of Genes and Genomes (KEGG) database (Kanehisa and Goto, 2000). Then, each enzyme with an E.C. number was associated with its respective metabolic map. In a similar manner, for TFs we used the compendium of TFs predicted by Rivera-Gómez et al. (2017); assigned from the hidden Markov model (HMM) profiles. To determine the abundance and distribution of each dataset, an incidence rate of the genome and a heatmap for each genome were determined.

### Enrichment Analysis

To evaluate the functional association between the modules and TFs and enzymes, an enrichment analysis using a hypergeometric test was conducted. The resulting distribution thus describes the probability of finding *x* domains associated with a particular category in a list of interest *k*, from a set of *N* domains containing *m* domains that are associated with the same category. We set statistical significance at a *P*-value of <0.05. All analyses were performed in Python (https://www.python.org/).

### Similarity Analysis

To determine the similarity degree between the different enriched modules, orthologous proteins between each pair of genomes were identified. Orthologs were accepted if they had an *e*-value <1e-6, sequence identity >30%, and alignment length >60% of the individual proteins. Then, the Jaccard index was calculated for each pair of modules, which is defined as the size of the intersection that represents the orthologs between each pair of modules of two organisms, divided by the union size of the sample sets.

### Functional Annotation Analysis

To identify the biological process in each module, we used the Database for Annotation, Visualization and Integrated Discovery (DAVID; http://david.abcc.ncifcrf.gov/), which is a gene functional classification system that integrates a set of functional annotation tools (Huang et al., 2009).

## Results and Discussion

### Construction of Gene Co-expression Networks

**Figure 1:**
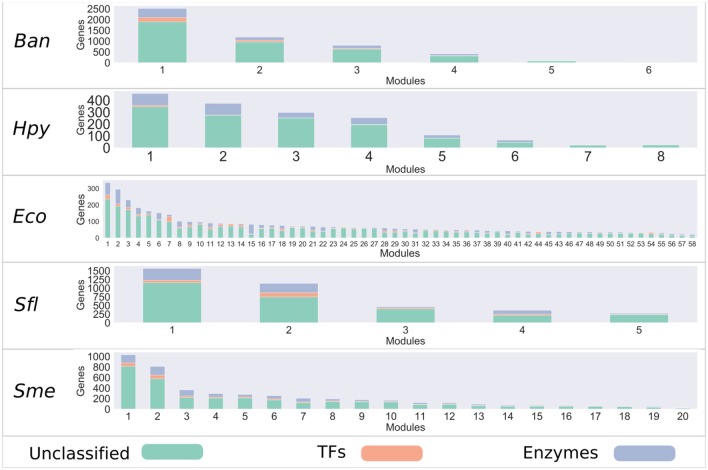
Bacteria co-expression modules. On the x-axis are shown the modules identified with the WGCNA package, identified with a number. The distribution of modules is represented in decreasing order, where the y-axis represents the number of genes per module. Each module is made up of a set of genes associated with TFs (orange), metabolic enzymes (blue), and unclassified genes (green).

In order to determine which genes share similar co-expression patterns in bacteria, a set of co-expression networks was inferred for 17 different bacteria with WGCNA R package (Largfelder and Holvarth, 2008), based on the information deposited in the COLOMBOS database (Moretto et al., 2016). We considered signed networks, because this method takes into account the sign of the underlying correlation coefficient and it has been shown that these networks can identify modules with more significant enrichment of functional groups (Medina and Lubovac-Pilav, 2016; Liu et al., 2018). Based on this approach, the reconstructed co-expression networks had a coverage of around 90% of the predicted open reading frames (ORFs) for each of the bacteria analyzed. In addition, modules inferred showing different sizes, for instance, *Escherichia coli* (Eco) contains the highest number of modules with 58, while for *Shigella flexneri* (Sfl) only 5 modules were identified (see Figure 1 and Table S1 and Figure S2).

It has been described that, i.e., more samples usually lead to more robust and refined results (Horvath, 2011). However, in the case of the dataset used in our study, the number of samples did not reflect the number of Gene Expression Omnibus (GEO) series used for each bacterium, and this would have influenced the number of modules identified for each organism, as in the case of *Bacillus anthracis* strain Ames (Ban), for which the samples belonged to 4 GEO series, or *Helicobacter pylori* 26695 (Hpy), for which the samples belonged to 8 GEO series, while *Salmonella enterica* LT2 (Stm) samples came from 16 GEO series.

### Highly Enriched Modules in TFs and Metabolism Terms

Two processes highly conserved between all the organisms are metabolism and gene regulation, which are mediated by enzymes that catalyze metabolic reactions and by DNA-binding TFs, respectively (Browning and Busby, 2004; Peregrín-Alvarez et al., 2009). In order to identify if metabolism and regulation-related genes share similar co-expression patterns, their distributions into the modules were mapped. Therefore, a collection of TFs, which were identified by homology from a dataset compendium of TFs previously characterized together with family-specific HMM profiles, as well as a compendium of metabolic enzymes of the KEGG repertoire for each one of the 17 bacteria, was used to integrate the information for the inferred modules.

We found that both enzymes and TFs are distributed in almost every co-expression module. This finding is consistent with previous works on modules of co-expression of *E. coli*, where TFs are distributed in all the modules, which allows them to be regulated (Sastry et al., 2019). However, there are modules that have a greater proportion of TFs or enzymes, and this leads us to think that some modules may be more relevant than others in the context of gene regulation or metabolism (Figure 1).

**Figure 2:**
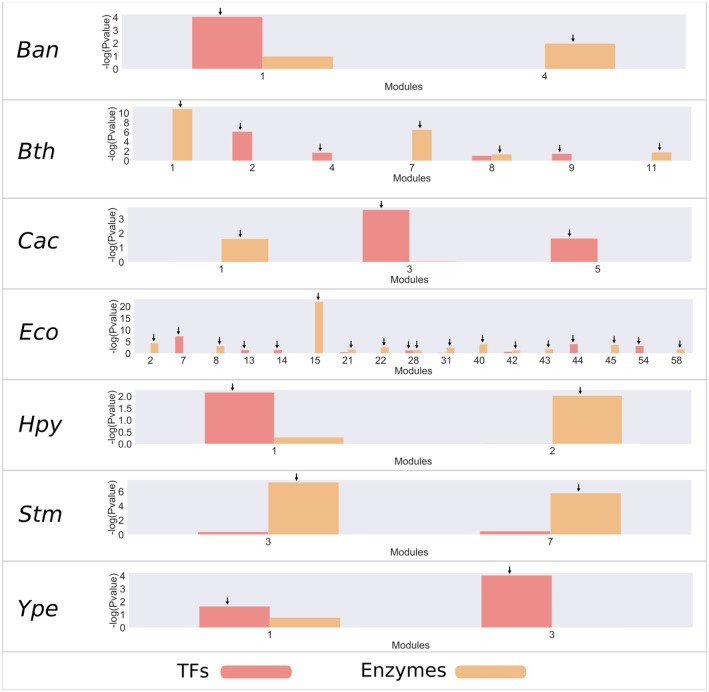
Enrichment of TFs and metabolic enzymes. Modules with a –log10 (*P*-value) >1.5 (corresponding to a *P*-value <0.05) were selected as enriched and are indicated by an arrow on the bar. The red bars represent modules enriched with TF families, and the orange bars represent modules enriched with enzymes.

To identify relevant modules that consider those regulatory mechanisms and metabolism, an analysis of enrichment was carried out by using a hypergeometric test with the set of TFs and the enzymes associated with metabolism for each of the modules (Figure 2 and Figure S3). From this analysis, we found that most bacteria have an average 2 modules enriched with TFs, with the exception of *E. coli* K-12 MG1655 (Eco), which has 11 modules enriched, and *S. enterica* LT2 (Stm), which does not contain modules enriched with TFs. On the other hand, bacteria contain an average of 4 modules enriched for metabolic enzymes; where *E. coli* is the only species with more modules, with 17. In contrast, *Yersinia pestis* (Ype) does not contain modules enriched with metabolic enzymes.

The most enriched modules with TFs contain on average 27% of the predicted genes with this function. Meanwhile, the modules enriched with metabolic enzymes contain on average 19% genes predicted to be related to metabolism in each organism. Specifically, *B. anthracis* strain Ames (Ban), *H. pylori* 26695 (Hpy), and *S. flexneri* 301 (Sfl) contain around 50% of all predicted TFs. In the same way, *Bacteroides thetaiotaomicron* VPI-5482 (Bth), *Clostridium acetobutylicum* ATCC824 (Cac), *Lactobacillus rhamnosus* GG (Lrh), and *Sinorhizobium meliloti* (Sme) modules contain around 30% of the genes associated with metabolic enzymes.

Based on the modules identified, diverse and interesting findings emerged, such as the fact that there is at least one module with a high percentage of TFs and enzymes, and this led us to evaluate if the richer modules also have a preference for a particular TF family or metabolic maps.

### TFs and Metabolism Terms More Abundant

**Figure 3:**
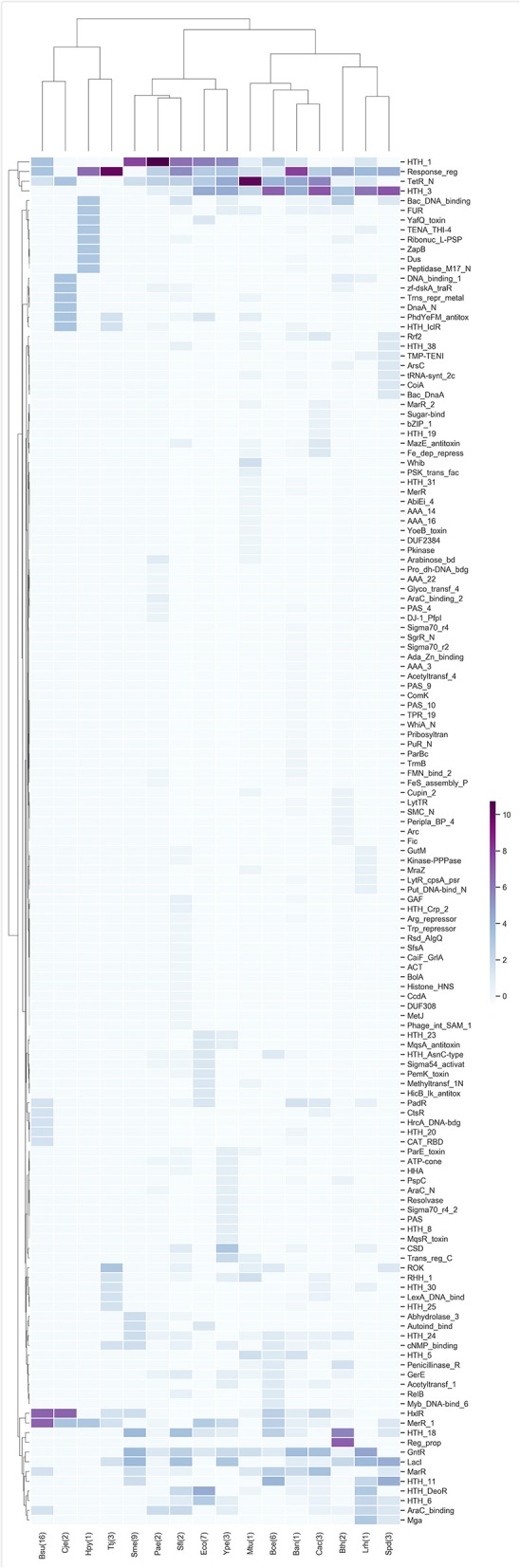
TF families identified as frequent in the enriched modules. *Z*-score hierarchical clustering based on Euclidean distance measure and Ward's method for linkage analysis. Each row represents the PFAM and each column represents the most enriched module for that bacterial species.

The TFs of each of the highest enrichment modules were classified using the families described in the PFAM database, and the z-scores of the frequency of the families were clustered hierarchically based on Euclidean distance measure and Ward's method for linkage analysis. We determined that the families most frequently present in these modules belong to Response_reg, LysR (HTH_1), Cro-C1 (HTH_3), TetR_N, and GntR (Figure 3), and these findings are in agreement with previous results for families more abundant in bacteria (Perez-Rueda et al., 2018).

In this regard, the Response_reg family is related to the two-component systems of bacteria, in which a signal is received from a sensor protein (i.e., the two components). This family of regulators allows the organism to adapt to a wide range of environments, stressors, and growth conditions (Skerker et al., 2005). Another family identified in the modules corresponds to TetR_N, which was one of the most abundant within our study; it is involved in regulating antibiotic resistance, catabolic pathways, biosynthesis of antibiotics, osmotic stress response and pathogenicity. These regulators typically function as repressors (Ramos et al., 2005; Cuthbertson and Nodwell, 2013).

Other families of regulators identified as abundant in the modules were LysR (HTH_1), a family of TFs involved in the regulation of a wide variety of processes that includes the regulation of amino acid biosynthesis and catabolism, stress responses and cell detoxification (Maddocks and Oyston, 2008); and Cro-C1 (HTH_3), which is part of the binary switch that regulates lytic/lysogenic growth of phages by differential binding to the operator sites (Steinmetzer et al., 2002).

In *Bacillus subtilis* 168 (Bsu) and *Campylobacter jejuni* NCTC 11168 (Cje), the abundant families are HxlR, which includes activators involved in the detoxification of formaldehyde, and MerR_1, which responds to environmental stimuli, such as heavy metals, oxidative stress or antibiotics and a subgroup of transcription activators that respond to metal ions (Brown et al., 2003). Meanwhile, in *B. thetaiotaomicron* VPI-5482 (Bth) the most abundant families are HTH_18, which is related to the arabinose operon regulatory protein AraC (Gallegos et al., 1993), and Reg_prop, which is part of a hybrid two-component system and are a key part of this species' ability to sense and degrade complex carbohydrates in the gut (Lowe et al., 2012).

**Figure 4:**
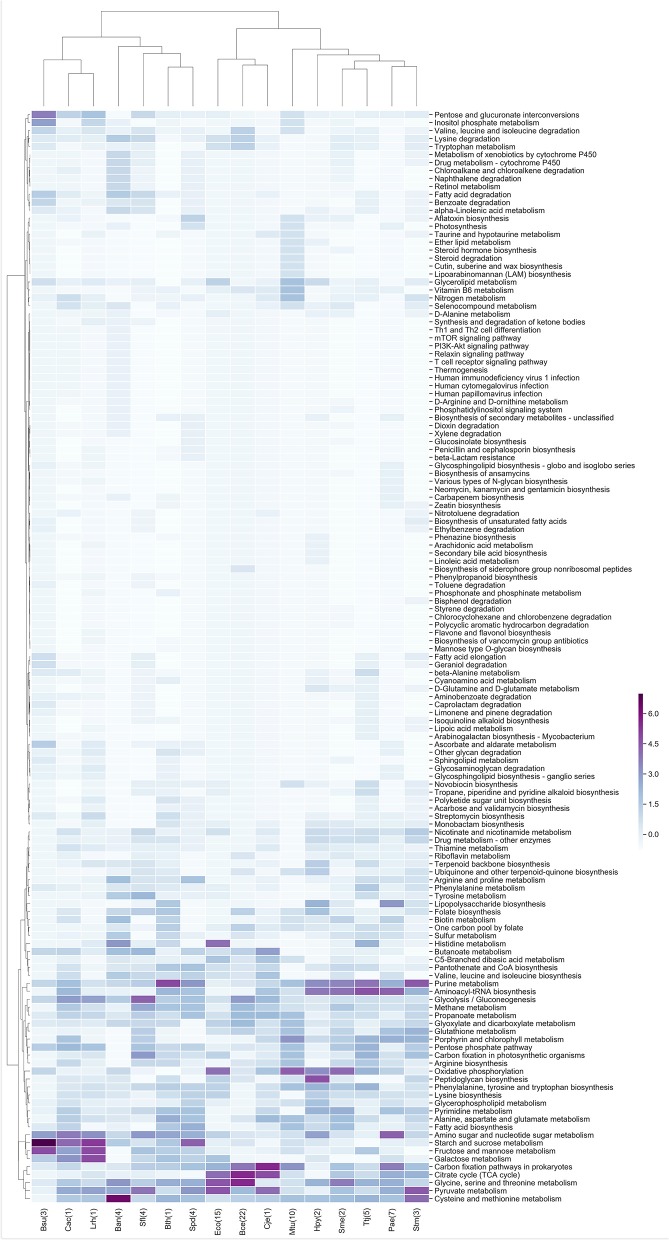
Metabolic maps more frequent in the enriched modules. *Z*-score hierarchical clustering based on Euclidean distance measure and Ward's method for linkage analysis. Each row represents a metabolic map (KEGG), and each column represents the most enriched module, with E.C. numbers for each species.

In the same context, the metabolic enzymes were classified according to the KEGG maps, and the z-scores of the frequency of each metabolic map were clustered, similar to our groupings for TF families. In general, we identified that the central metabolism pathways that includes glycolysis/gluconeogenesis, the citrate cycle (TCA cycle) and pyruvate metabolism are expressed independently of the experimental conditions analyzed, similar to the case for nucleotide metabolism. Another conserved cluster is related to carbohydrate metabolism and includes amino sugar and nucleotide sugar metabolism, starch and sucrose metabolism, galactose metabolism, fructose and mannose metabolism and pentose and glucuronate interconversions (Figure 4).

In Figure 4, there are well-defined clusters, such as the one in *B. anthracis* str. Ames (Ban) that contains maps belonging to xenobiotic biodegradation and metabolism of xenobiotics by cytochrome P450 and to drug metabolism by cytochrome P450, which is mediated by a class II P450 system in this organism (De Mot and Parre, 2002). In addition, in *Mycobacterium tuberculosis* H37Rv (Mtu) we identified maps related to glycerolipid metabolism, which is used to generate glycerols from the host's fatty acids, the vitamin B6 metabolic pathway, which is essential for survival and virulence (Dick et al., 2010), and a nitrogen metabolic pathway that is essential for growth and virulence of this bacterium (Gouzy et al., 2014).

In summary, we identified diverse families of TFs and metabolic maps common to all modules in the organisms analyzed, suggesting that common regulatory processes governing a large diversity of metabolic genes expressed under different conditions, and by consequence the global response could be similar even when the organisms employ a diverse repertoire of genes, i.e., not homologous genes. This led us to evaluate the similarity between these modules.

### Metabolism and Similar Regulation

**Figure 5:**
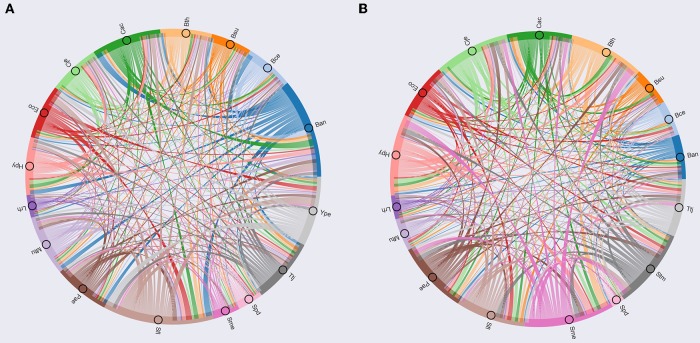
Circos based in Jaccard index. **(A)** Circos based on TFs; **(B)** Circos based on metabolic maps.

To determine the organisms with similar regulation, we calculated the Jaccard index between each pair of modules enriched with TFs, using the number of orthologs shared between each pair of organisms, additionally each module was analyzed by means of Gene Ontology using DAVID (Huang et al., 2009). The Jaccard index matrix was used to build a circos plot (Figure 5A), showing similar modules between *S. flexneri* 301 (Sfl), *B. anthracis* (Ban), and *Y. pestis* C092 (Ype), which are characterized as having genes related to biosynthetic process, regulation of cellular process and regulation of primary metabolic processes.

The second group contains *Pseudomonas aeruginosa* PAO1 (Pae), *B. thetaiotaomicron* VPI-5482 (Bth), *M. tuberculosis* H37Rv (Mtu), *Thermus thermophilus* HB8 (Ttj), *C. acetobutylicum* ATCC824 (Cac), *E. coli* K-12 MG1655 (Eco), which include gene related to regulation of cellular and metabolic process, single-organism localization and cellular process and regulation of metabolic process. Finally, the third group consists of *Bacillus cereus* ATCC14579 (Bce), *H. pylori* 26695 (Hpy), *C. jejuni* NCTC 11168 (Cje), *B. subtilis* 168 (Bsu), *L. rhamnosus* GG (Lrh), *Streptococcus pneumoniae* D39 (Spd), *S. meliloti* 1021 (Sme), which have gene related to regulation of cellular process, single-organism metabolic process and nitrogen compound metabolic process.

On the other hand, in the modules related to metabolism, we used the Jaccard index between each pair of modules enrichment with enzymes to identify the similar modules (Figure 5B). Based on this approach, we identified that *S. meliloti* 1021 (Sme) is a module that contains a high proportion of orthologs with the other modules, where genes related to cellular metabolic process, primary metabolic process, nitrogen compound metabolic process and organism substance metabolic process were identified. This result could be associated to the prevalence of genetic redundancy in this bacterium, an in particular to those genes involved in a variety of metabolic pathways, including central carbon metabolism, transport, and amino acid biosynthesis (diCenzo and Finan, 2015); and the number of genes with some regulatory mechanisms identified in one of the three replicons, and the function of regulated genes was found to be in accordance with the overall replicon functional signature: house-keeping functions for the chromosome, metabolism for the chromid, and symbiosis for the megaplasmid (Galardini et al., 2015).

This group include *C. jejuni* NCTC 11168 (Cje), *B. thetaiotaomicron* VPI-5482 (Bth), *S. enterica LT2* (Stm), *P. aeruginosa PA01* (Pae), *C. acetobutylicum* ATCC824 (Cac), *H. pylori* 26695 (Hpy), *S. flexneri* 301 (Sfl), which are characterized by genes related to cellular metabolic process, single-organism cellular process, biosynthetic process and organic substance metabolic process. Finally, this group includes *E. coli* K-12 MG1655 (Eco), *B. anthracis* strain Ames (Ban), *T. thermophilus* HB8 (Ttj), *B. subtilis* 168 (Bsu), *M. tuberculosis* H37Rv (Mtu), *B. cereus* ATCC 14579 (Bce), *L. rhamnosus* GG (Lrh), *S. pneumoniae* D39 (Spd); these species have gene related to catabolic process, single-organism metabolic process, and establishment of localization.

In addition, enriched modules were analyzed to determine those genes with greater connectivity. To this end, we used the first 100 nodes that most correlate in each module where the identified genes had the highest connectivity or highest node degree, which describes the number of interactions or edges adjacent to the node (Table S2). Many of the most highly connected nodes are related to nitrogen compound metabolic process, biosynthetic process, cellular metabolic process, primary metabolic process, and single-organism metabolic process, although in some cases the most important hub genes encode for hypothetical proteins, which would allow future analysis to determine their functional role.

**Figure 6:**
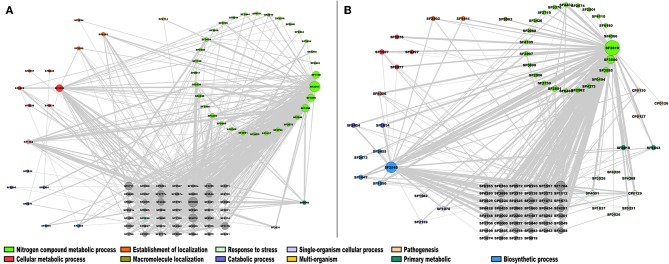
Co-expression network of *S. flexneri*. The most highly correlated genes were plotted in Cytoscape (Smoot et al., 2010). The size of the modules corresponds to their degree of connectivity, while the widths of the edges represent the weights of the correlations, gray nodes do not have an assigned function. **(A)** TFs; **(B)** metabolism modules.

From this analysis, in the case of the module 2 enriched with TFs of *S. flexneri* 301 (Sfl), the most highly connected genes were SF2819, an activator of the L-fucose operon from the DeoR family, and SF2545, a polyphosphate kinase [E.C. 2.7.4.1] involved in the nitrogen compound metabolic process and biosynthetic process, respectively; in addition, two hypothetical proteins, SF1784 and SF3500 were also identified as highly connected genes (Figure 6A). In module 4, that was enriched with enzymes, the genes with the highest connectivity were SF2911, which encodes a phosphoglycerate kinase [E.C. 2.7.2.3] involved in nitrogen compound metabolic process; SF0929, which encodes an aminopeptidase N [E.C. 3.4.11.2] involved in the Glutathione metabolism; and SF4274, a NAD(P)H dehydrogenase (quinone) [EC:1.6.5.2] involved in Metabolic pathways (Figure 6B). This result correlates with the fact that glutathione and quinone metabolism play a major role in the defense against redox cycling-derived oxidative stress (Kelly et al., 2019), reinforcing the notion that common expression patterns identified in this work correlates with similar protein roles in the cell.

**Figure 7:**
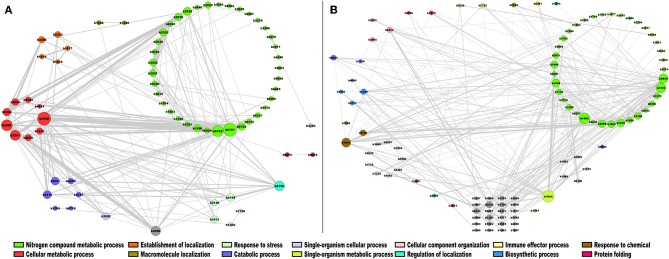
Co-expression network of *E. coli*. The most highly correlated genes were plotted in Cytoscape (Smoot et al., 2010). The sizes of the modules correspond to their degrees of connectivity, while the widths of the edges represent the weights of the correlations, gray nodes do not have an assigned function. **(A)** TFs; **(B)** metabolism modules.

In the case of module 7 enriched with TFs in *E. coli*, we identified the following genes with the highest connectivity: *ydgJ* (b1624), a probable D-galactose 1-dehydrogenase, involved in single-organism metabolic process (Reed et al., 2003); *ribC* (b1662) (for riboflavin synthase), which catalyzes the final step in riboflavin biosynthesis (Eberhardt et al., 1996); *ogt* (b1335), which encodes a methyltransferase enzyme for the repair of alkylated DNA (Taira et al., 2013); and *deoR* (b0840), which is involved in the negative expression of genes related to transport and catabolism of deoxyribonucleoside nucleotides (Garces et al., 2008). These highly correlated genes are mainly involved in biosynthetic processes and nitrogen compound metabolic processes, as shown in Figure 7A. In this regard, DeoR and regulated genes have been involved in DNA damage response by drugs, modifying the nucleotide level modulation (Sangurdekar et al., 2011), suggesting that b1335 and b0840 are functionally closer. Therefore, the other genes identified in this module could also participate in a similar response, however further evidence is necessary. On the other hand, in module 15, which is enriched with enzymes, the genes with the highest connectivity were *sucB* (b0727), *sucC* (b0728), and *sucD* (b0729), which are associated with the citrate cycle, an important aerobic pathway for the final steps of the oxidation of carbohydrates and fatty acids (Buck et al., 1986); *nuoH* (b2282), *nuoI* (b2281), *nuoJ* (b2280), and *nuoG* (b2283), involved in the oxidative phosphorylation pathway (Bongaerts et al., 1995) (Figure 7B).

## Conclusions

In this work, we identified and analyzed modules considered relevant from a metabolic and regulatory point of view in a set of bacteria, using a weighted gene co-expression analysis method. Based on this analysis, we identified some modules enriched with TFs and metabolic enzymes. In the case of regulation, we identified TFs from the families Response_reg, TetR_N, LysR, and HTH_3, which are mainly related to biological processes, such as biosynthetic processes, cellular metabolic processes, nitrogen compound metabolic processes and primary metabolic processes. On the other hand, the modules enriched with enzymes are associated mainly with primary metabolic, organic substance metabolic, cellular metabolic and nitrogen compound metabolic processes. Our approach also identified genes with similar expression patterns and involved in similar metabolic or regulatory roles, such as DeoR and Ogt. In summary, this analysis allowed us to determine that, despite the diversity of experimental information available for each organism, these mechanisms are similar in all of the organisms, and this will allow us to address new experimental results, such as the use of gene expression data in metagenomic studies.

## Data Availability Statement

All datasets generated for this study are included in the article/Supplementary Material.

## Author Contributions

EG-V performed the experiments, analyzed the data, and wrote the paper. EP-R analyzed the data and wrote the paper.

### Conflict of Interest

The authors declare that the research was conducted in the absence of any commercial or financial relationships that could be construed as a potential conflict of interest.

## Supplementary Material

Supplementary Material S1 Dataset processed by PCA and goodSamplesGenes.

Supplementary Material S2 General collection of scripts to process, analyze, and visualize the dataset of previously processed.

Table S1 Collection of co-expression modules for each of the organisms.

Table S2 Collection of hub genes for each organism.

Figure S1 Workflow of the analysis procedure.

Figure S2 Co-expression modules for all organisms. On the x-axis are shown the modules identified with the WGCNA package, identified with a number. The distribution of modules is represented in decreasing order, where the y-axis represents the number of genes per module.

Figure S3 Enrichment of TFs and metabolic enzymes for all organisms. Modules with a –log10 (*P*-value) >1.5 (corresponding to a *P*-value <0.05) were selected as enriched and are indicated by an arrow on the bar.
