# Supplementary figures and images for: Identification of Modules With Similar Gene Regulation and Metabolic Functions Based on Co-expression Data
Source: Front Mol Biosci. 2019 Dec 13;6:139. doi: 10.3389/fmolb.2019.00139 (PMC6929668; doi:10.3389/fmolb.2019.00139)

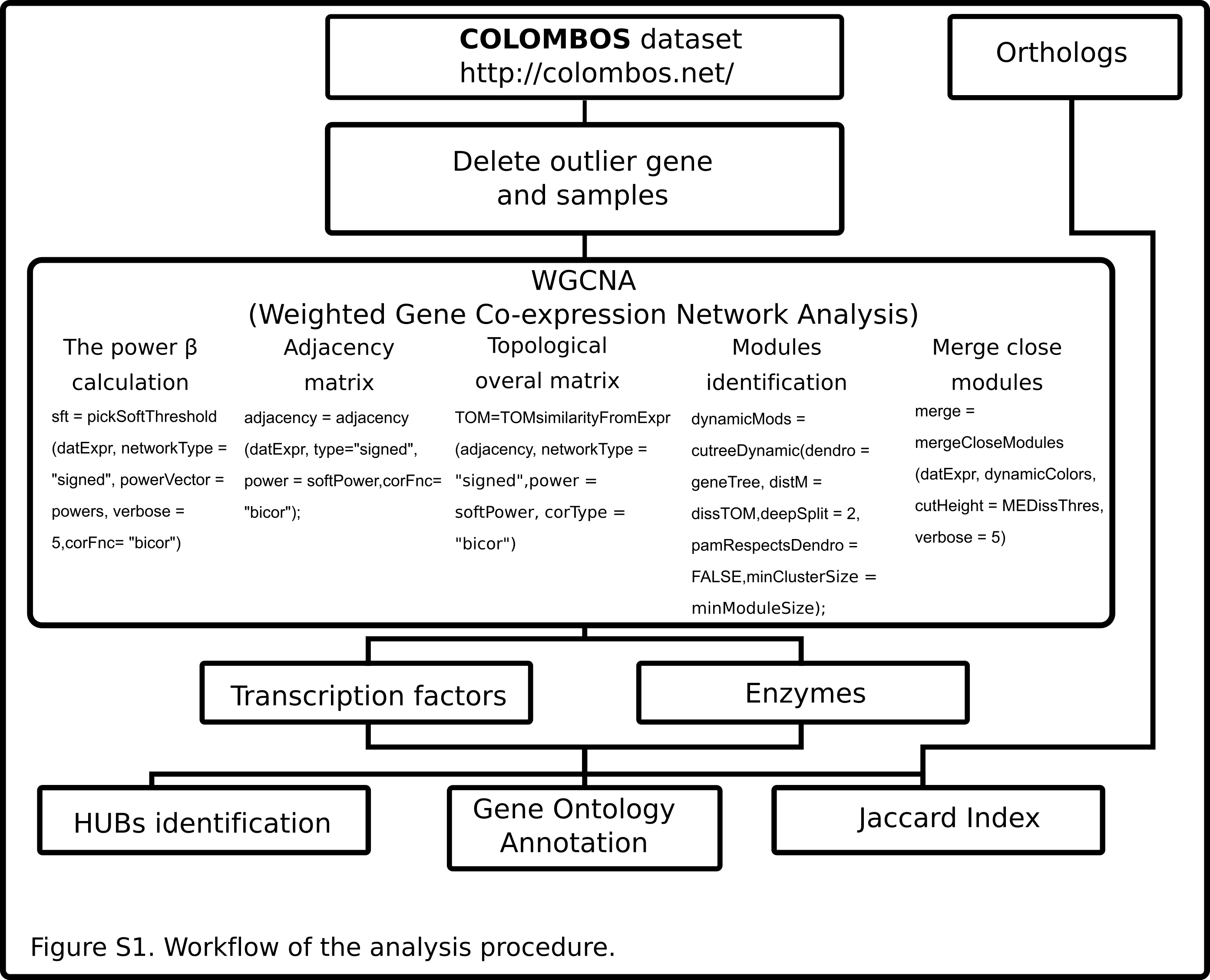

Supplement: Figure S1 — Workflow of the analysis procedure. [file Image_1.png]

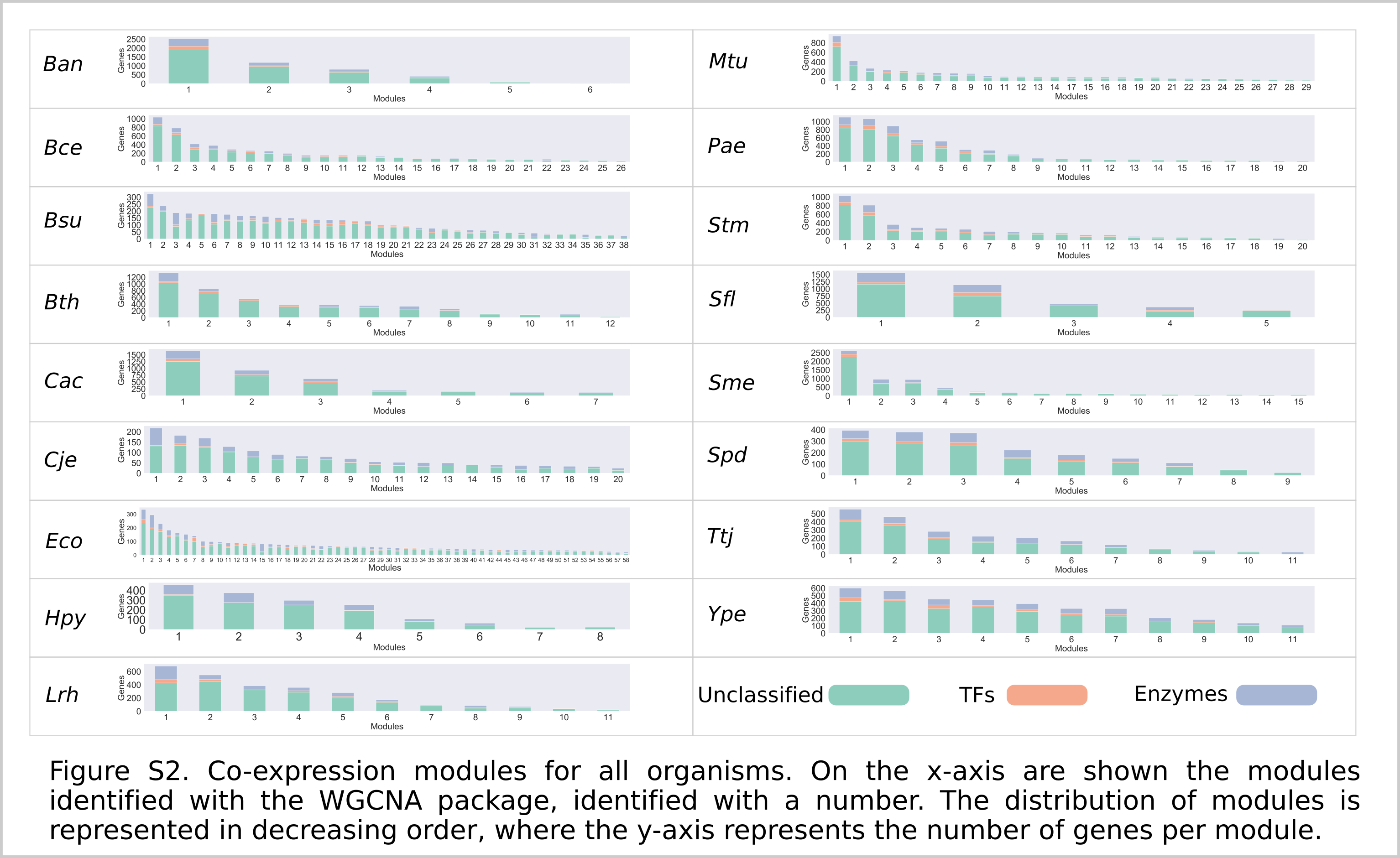

Supplement: Figure S2 — Co-expression modules for all organisms. On the x-axis are shown the modules identified with the WGCNA package, identified with a number. The distribution of modules is represented in decreasing order, where the y-axis represents the number of genes per module. [file Image_2.png]

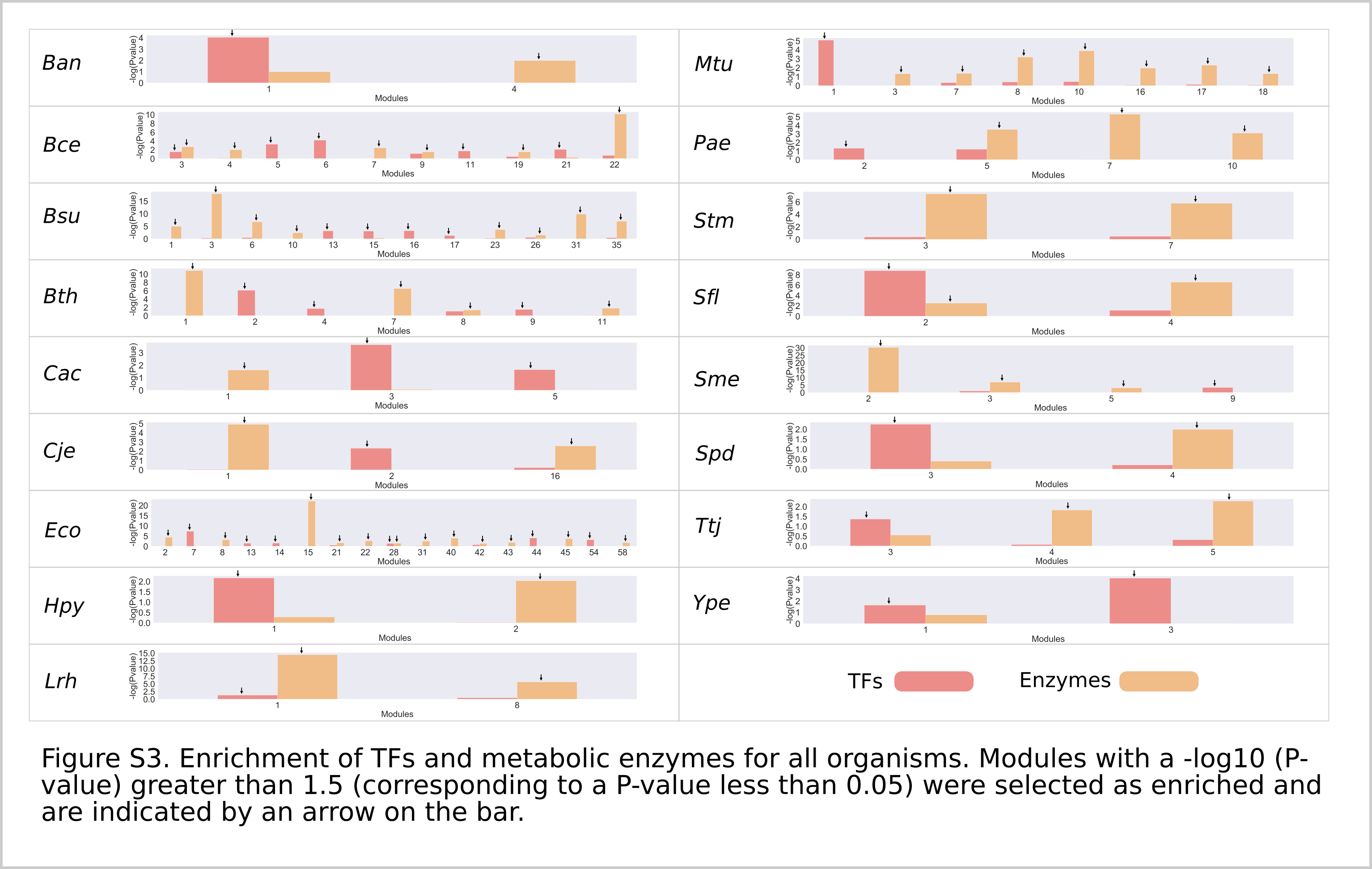

Supplement: Figure S3 — Enrichment of TFs and metabolic enzymes for all organisms. Modules with a –log10 (P-value) >1.5 (corresponding to a P-value <0.05) were selected as enriched and are indicated by an arrow on the bar. [file Image_3.png]
